# Gestational diabetes modifies the association between PlGF in early pregnancy and preeclampsia in women with obesity

**DOI:** 10.1016/j.preghy.2018.07.003

**Published:** 2018-07

**Authors:** Matias C. Vieira, Shahina Begum, Paul T. Seed, Dania Badran, Annette L. Briley, Carolyn Gill, Keith M. Godfrey, Deborah A. Lawlor, Scott M. Nelson, Nashita Patel, Naveed Sattar, Sara L. White, Lucilla Poston, Dharmintra Pasupathy, UPBEAT Consortium

**Affiliations:** a Department of Women and Children’s Health, School of Life Course Sciences, Faculty of Life Sciences and Medicine, King’s College London, London SE1 7EH, UK; b Núcleo de Formação Específica em Ginecologia e Obstetrícia, Escola de Medicina, Pontifícia Universidade Católica do Rio Grande do Sul, Porto Alegre 90610-000, Brazil; c Faculty of Medicine, Imperial College London, London SW7 2AZ, UK; d NIHR Biomedical Research Centre at Guy’s & St Thomas’ NHS Foundation Trust and King’s College London, London SE1 7EH, UK; e MRC Lifecourse Epidemiology Unit and NIHR Southampton Biomedical Research Centre, University of Southampton and University Hospital Southampton NHS Foundation Trust, Southampton SO16 6YD, UK; f MRC Integrative Epidemiology Unit at the University of Bristol and School of Social and Community Medicine, University of Bristol, Bristol BS8 2BN, UK; g NIHR Bristol Biomedical Research Centre at University Hospitals Bristol NHS Foundation Trust and University of Bristol, Bristol BS8 2BN, UK; h School of Medicine, University of Glasgow, Glasgow G31 2ER, UK; i Institute of Cardiovascular and Medical Sciences, University of Glasgow, Glasgow G12 8TA, UK

**Keywords:** BMI, body mass index, FDR, False discovery rate, GDM, gestational diabetes mellitus, HbA1c, hemoglobin A1c, HDL, high density lipoprotein, Hs-CRP, high sensitivity C Reactive Protein, IL-6, interleukin-6, OGTT, oral glucose tolerance test, PlGF, placental growth factor, sFlt-1, soluble VEGF receptor, VEGF, vascular endothelial growth factor, Obesity, Preeclampsia, Gestational diabetes mellitus, Risk factors, Biomarkers

## Abstract

**Objective:**

To identify clinical and biomarker risk factors for preeclampsia in women with obesity and to explore interactions with gestational diabetes, a condition associated with preeclampsia.

**Study design:**

In women with obesity (body mass index ≥ 30 kg/m^2^) from the UK Pregnancies Better Eating and Activity Trial (UPBEAT), we examined 8 clinical factors (socio-demographic characteristics, BMI, waist circumference and clinical variables) and 7 biomarkers (HDL cholesterol, hemoglobin A1c, adiponectin, interleukin-6, high sensitivity C-reactive protein, and placental growth factor (PlGF)) in the early second trimester for association with later development of preeclampsia using logistic regression. Factors were selected based on prior association with preeclampsia. Interaction with gestational diabetes was assessed.

**Main outcome measure:**

Preeclampsia.

**Results:**

Prevalence of preeclampsia was 7.3% (59/824). Factors independently associated with preeclampsia were higher mean arterial blood pressure (Odds Ratio (OR) 2.22; 95% Confidence Interval (CI) 1.58–3.12, per 10 mmHg) and lower PlGF (OR 1.39; 95% CI 1.03–1.87, per each lower 1 log2). The association of PlGF with preeclampsia was present amongst obese women without gestational diabetes (OR 1.91; 95% CI 1.32–2.78), but not in those with GDM (OR 1.05; 95% CI 0.67–1.63), p = 0.04 for interaction.

**Conclusion:**

The relationship between PlGF and preeclampsia differed in women with obesity according to gestational diabetes status, which may suggest different mechanistic pathways to preeclampsia. Whilst replication is required in other populations, this study suggests that performance of prediction models for preeclampsia should be confirmed in pre-specified subgroups.

## 1 Introduction

Globally, preeclampsia is one of the principal causes of maternal and neonatal morbidity and mortality, with a prevalence of 3–5% in unselected populations [1]. The principal feature of preeclampsia is new onset hypertension with proteinuria after 20 weeks’ gestation. In the absence of curative treatment, management involves stabilization of the mother and fetus, followed by timely delivery. The cause of preeclampsia remains unclear but certain co-morbidities predispose women to an increased risk, including gestational diabetes mellitus (GDM) and obesity, the focus of the present study [2], [3].

Obesity is increasing worldwide. By 2025, it is expected that one in every five women of reproductive age across the world will have obesity of whom at least 7–9% are likely to develop preeclampsia [4]. Some reports suggest an associated increase in the prevalence of preeclampsia, however other risk factors, such as increasing maternal age and a reduction in smoking, may have contributed [5]. Insulin resistance and a mild inflammatory state have been implicated in the pathophysiology of preeclampsia in women with obesity, which could also explain the association between GDM and preeclampsia [3], [6], [7]. However, in a recent study we found no evidence of a link between insulin resistance or inflammation biomarkers and preeclampsia in a group of nulliparous women with obesity [8]. As women with obesity who develop GDM have an exaggerated insulin resistance and inflammation biomarker profile [9], we hypothesized that the association of insulin resistance and inflammatory biomarkers with preeclampsia may be confined to women who develop GDM.

The aim of this study was to identify clinical and biomarker risk factors for preeclampsia in women with obesity and to explore interaction with GDM status. This was undertaken as a secondary analysis of a large cohort of pregnant women with obesity, the UK Pregnancies Better Eating and Activity Trial (UPBEAT), from whom a detailed clinical history had been obtained and blood samples taken in the early second trimester, prior to the development of preeclampsia [10].

## 2 Materials and methods

This study was a prospective cohort using data from UPBEAT, a randomized controlled trial of a complex behavioral intervention aimed at reducing GDM and large for gestational age infants [10]. UPBEAT recruited pregnant women with obesity (body mass index (BMI) ≥30 kg/m^2^) with singleton pregnancies at 15^+0^–18^+6^ weeks’ gestation. Women were excluded if they had underlying disorders, including pre-pregnancy diagnosis of essential hypertension, diabetes, renal disease, systemic lupus erythematosus, antiphospholipid syndrome, sickle cell disease, thalassaemia, coeliac disease, thyroid disease, and current psychosis; or if they were currently being prescribed metformin. Detailed information was collected at trial entry (15^+0^–18^+6^ weeks gestation) on maternal clinical characteristics, including socio-demographic information and anthropometric measures; non-fasting blood samples were also taken. Extensive information on maternal and infant outcomes was recorded. Research ethics committee approval was obtained (UK Integrated Research Application System, reference 09/H0802/5) and all women provided written consent.

For the purpose of the present study, women with incomplete information on preeclampsia status, oral glucose tolerance test (OGTT) results, clinical and biomarker data were excluded. The UPBEAT intervention was not associated with a difference in the prevalence of preeclampsia or GDM [10], we therefore treated the study population as a cohort for the purpose of this analysis.

The primary outcome of the present study was preeclampsia which was defined according to the International Society for the study of Hypertension in Pregnancy (ISSHP) criteria: two measures of systolic (≥140 mmHg) or diastolic blood pressure (≥90 mmHg) taken at least four hours apart and the presence of proteinuria (spot urine protein/creatinine ≥30 mg/mmol [0.3 mg/mg] or ≥300 mg/day or at least 1 g/L [‘2 + ’] on dipstick testing) [11]. For the purpose of this study, preeclampsia diagnosis was reviewed based on blood pressure and proteinuria values recorded by the research team. GDM, the pre-specified interaction analysis, was assessed by a universal screening 75 g OGTT and diagnosis was based on the International Association of Diabetes and Pregnancy Study Groups (IADPSG) criteria comprising one or more positive plasma glucose values: fasting ≥5.1 mmol/l, 1 h ≥10.0 mmol/l, 2 h ≥8.5 mmol/l [12], [13].

Potential clinical risk factors included 8 variables selected on the basis of previously identified associations with preeclampsia and ability to translate to clinical practice. These were: age, nulliparity, BMI, waist circumference, known history of preeclampsia, first degree family history of hypertension and mean arterial blood pressure. Maternal sum of skinfold thicknesses (derived from the sum of mean triceps, biceps, subscapular, and suprailiac measurements made by trained research staff) was also explored due to its known association with BMI. Blood pressure was recorded using the pregnancy validated Microlife BP3BT0-A blood pressure monitor (Microlife, Widnau, Switzerland) and maternal skinfold thicknesses (triceps, biceps, suprailiac and subscapular) were measured in triplicate, using Harpenden skinfold Calipers (Holtain Ltd, Felin-y-Gigfran, Crosswell, UK) [14]. Seven biomarkers were selected based on either a proposed role in preeclampsia pathogenesis in women with obesity, or a previously reported association with preeclampsia in weight heterogeneous women. These were: triglycerides, high-density lipoprotein (HDL), hemoglobin A1c (HbA1c), adiponectin, interleukin-6 (IL-6), high sensitivity C Reactive Protein (hs-CRP), and placental growth factor (PlGF). Biomarker measurement methodology is described in the Appendix (Table A1).

A complete case analysis was undertaken and we compared socio-demographic characteristics between the study population and women excluded from this analysis because of missing data. Assumptions for normality were checked for all predictors. Candidate biomarkers that showed positive skew were log transformed (triglycerides, HDL, adiponectin, IL-6, CRP, and PlGF). Transformation into log_2_ were performed so that odds ratios showed the effect associated with doubling the concentration. As low values of PlGF, are associated with preeclampsia, the scale was reversed, so that the odds ratio (OR) represented the increase in odds of preeclampsia associated with a reduction in PlGF. Clinical factors and biomarkers did not vary with gestational age at sampling, so additional correction for gestational age was not performed.

Descriptive statistics were presented as means (SD), median (IQR) and frequency of observations (percentages), as appropriate. Comparisons of socio demographic characteristics and pregnancy outcomes between women with and without preeclampsia were performed using Chi-square tests, Wilcoxon rank-sum tests and t-tests as appropriate. Univariable logistic regression was used to investigate the association between clinical risk factors and biomarkers and the risk of development of preeclampsia later in pregnancy. Assessment of biomarkers was corrected for multiple testing using the False Discovery Rate (FDR) [15]. Significant factors were identified if p < 0.05 (FDR adjusted p-value for biomarkers) and were combined in a final multiple regression model for preeclampsia. To address the hypothesis that risk factors for preeclampsia may differ by GDM status, we explored the effect of pre-specified factors (PlGF, IL-6, CRP) and any significant risk factor in multivariable analysis on preeclampsia separately in women with and without GDM (stratified analysis). Likelihood ratio tests were then used to perform formal tests of interaction. Sensitivity analyses were performed using multiple imputation by chained equations to assess the potential for selection bias in complete case analysis. Outcomes (preeclampsia or GDM) were not imputed and women with missing outcomes were excluded from sensitivity analysis. Statistical analysis was performed using Stata software, version 14.2 (StataCorp LP, College Station, Texas). This study has been reported in line with STROBE recommendations [16].

## 3 Results

**Fig. 1:**
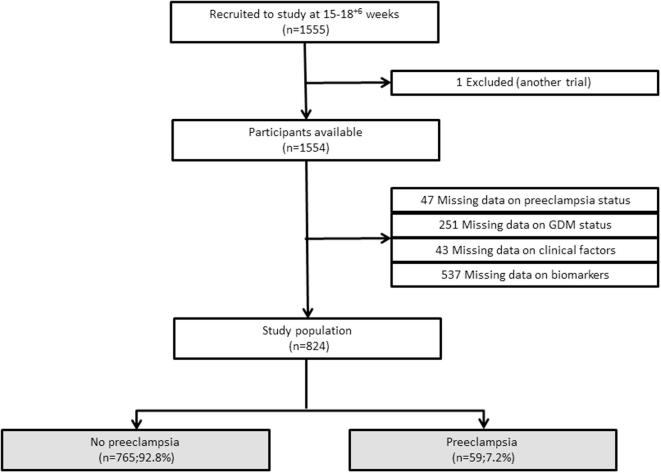
Study population.

Of the 1554 participants available for analysis, a total of 730 (47%) were excluded because of missing data for evaluation of preeclampsia diagnosis (n = 47), OGTT results (n = 251), clinical risk factors (n = 43), and biomarker measurement (n = 537) (Fig. 1). Although no difference was observed in age, BMI and smoking status, participants excluded from this analysis were more likely of black ethnicity and multiparous (Appendix; Table A2). The study population comprised 824 women; the prevalence of preeclampsia was 7.2% (59/824), including 21 de novo cases of preeclampsia after review of clinical data. The majority of cases presented at term (n = 49; 83.1%). The overall prevalence of preeclampsia was similar in all UPBEAT participants (6.3%).

Socio-demographic characteristics of women with and without preeclampsia are shown in Table 1. The average age of all women was 30.6 (±5.4) years. Women with preeclampsia had higher BMI in early pregnancy and were more likely to be nulliparous. Age, education, ethnicity and smoking status were similar between groups. Women with preeclampsia were more likely to have labor induced (62.7% *vs*. 34.8%, p < 0.01) and had a higher rate of preterm delivery (17.0% *vs.* 3.9%, p < 0.01), whilst their infants had lower median birth weight (3275 g; IQR 2700–3600 g *vs.* 3490 g; IQR 3168–3795 g, p < 0.01), lower Apgar scores (at 5 min < 7, 6.8% *vs*. 1.4%, p < 0.01), and were more likely to be admitted to neonatal intensive care unit (NICU) (18.6% *vs*. 7.2%, p < 0.01). Women with preeclampsia also had a higher rate of stillbirth or neonatal mortality (5.1% *vs*. 0.5%, p < 0.01). Amongst women with preeclampsia, the prevalence of any neonatal morbidity (combination of Apgar < 7, NICU admission or fetal/neonatal mortality) was 34.8% (8/23) in women with GDM compared to 13.9% (5/36) in women without GDM (p = 0.06).

**Table 1:** Socio-demographic characteristics and pregnancy outcomes according to preeclampsia status in women with obesity.

Variable	No preeclampsia Mean (SD) or n (%) n = 765	Preeclampsia Mean (SD) or n (%) n = 59	p value a
*Socio-demographic characteristics*			
Age	30.7 (5.4)	30.0 (4.9)	0.36
Body Mass Index b	35.0 (32.8–38.3)	37.6 (33.1–42.0)	0.01
Nulliparity	348 (45.5)	35 (59.3)	0.04
Full time education, ≥12 years	686 (89.7)	53 (89.8)	0.97
Ethnicity			
Asian	51 (6.7)	3 (5.1)	
Black	143 (18.7)	11 (18.6)	0.73
Other	40 (5.2)	5 (8.5)	
White	531 (69.4)	40 (67.8)	
Smoking at baseline	52 (6.8)	3 (5.1)	0.73

*Pregnancy outcomes*			
Induction of labor (n = 823)	266 (34.8)	37 (62.7)	<0.001
GA at delivery b, weeks (n = 823)	39.9 (38.9–40.9)	38.7 (37.7–39.7)	<0.001
Preterm delivery (n = 823)	29 (3.8)	10 (17.0)	<0.001
Birth weight b, grams (n = 823)	3490 (3170–3795)	3275 (2700–3600)	<0.001
Major PPH c (n = 821)	115 (15.1)	12 (20.3)	0.28
Mode of delivery (n = 823)			
LSCS in labor	136 (17.8)	10 (17.0)	
Operative vaginal	91 (11.9)	8 (13.6)	0.29
Prelabor LSCS	148 (19.4)	17 (28.8)	
Spontaneous vaginal	389 (50.9)	24 (40.7)	
Apgar < 7 at 5 min (n = 817)	11 (1.5)	4 (6.8)	0.003
NICU admission (n = 823)	55 (7.2)	11 (18.6)	0.002
Stillborn or neonatal death	4 (0.5)	3 (5.1)	<0.001

*Abbreviations:* GA – gestational age, IQR – interquartile range, LSCS – lower segment caesarean section, NICU – neonatal intensive care unit, PPH - postpartum hemorrhage, SD – standard deviation, wks – weeks.

a Comparisons performed using *t*-test or chi-squared test, as appropriate (unless otherwise stated).

b Results presented as median (IQR).

c Major PPH defined as estimated blood loss equal or above 1000 mls.

Clinical factors and biomarkers in women with and without preeclampsia are included in the Appendix (Table A3). Clinical factors associated with preeclampsia in univariable analyses were nulliparity (odds ratio (OR) 1.75; 95% Confidence Interval (CI) 1.02–2.99), BMI (OR 1.07; 95% CI 1.03–1.12, per kg/m^2^), higher sum of skinfolds (OR 1.01; 95% CI 1.00–1.02, per 1 mm), waist circumference (OR 1.03; 95% CI 1.01–1.05, per 1 cm) and mean arterial blood pressure (OR 2.43; 95% CI 1.06–1.13, per 10 mmHg) (Table 2). Correcting for multiple testing, reduced PlGF was the only biomarker associated with increased odds of developing preeclampsia (OR 1.46; 95% CI 1.10–1.94, per log_2_) in univariable analyses. In the multivariable regression, factors independently associated with preeclampsia were higher mean arterial blood pressure (OR 2.22; 95% CI 1.05–1.12, per 10 mmHg) and lower PlGF (OR 1.39; 95% CI 1.03–1.87, per log_2_) (Table 2).

**Table 2:** Clinical risk factors and biomarkers at 15^+0^–18^+6^ weeks’ gestation associated with preeclampsia in women with obesity (n = 824).

	Univariable Analysis OR (95% CI)	p value a	p value b	Multivariable Analysis OR (95% CI)	p value c
Age, years	0.98 (0.93–1.03)	0.36			
Nulliparous	1.75 (1.02–2.99)	0.04		1.50 (0.86–2.62)	0.15
BMI	1.07 (1.03–1.12)	0.002		1.04 (0.96–1.13)	0.29
Sum of skinfolds, mm	1.01 (1.00–1.02)	0.02		1.00 (0.99–1.02)	0.43
Waist, cm	1.03 (1.01–1.05)	0.01		1.00 (0.97–1.04)	0.96
Previous PE	2.53 (0.94–6.83)	0.07			
FH of hypertension	1.48 (0.87–2.51)	0.15			
MAP, per 10 mmHg	2.43 (1.75–3.37)	<0.001		2.22 (1.58–3.12)	<0.001
HDL, per log_2_ of mmol/l	0.48 (0.26–0.90)	0.02	0.05		
Triglycerides, per log_2_ of mmol/l	1.77 (1.02–3.08)	0.04	0.06		
HbA1c, mmol	1.07 (1.00–1.15)	0.04	0.07		
Adiponectin, per log_2_ of ug/ml	0.82 (0.62–1.09)	0.17	0.20		
IL-6, per log_2_ of pg/ml	1.46 (1.10–1.94)	0.008	0.06		
hs-CRP, per log_2_ of mg/L	1.15 (0.90–1.46)	0.27	0.27		
PlGF d, per log_2_ of pg/ml	1.46 (1.10–1.94)	0.01	0.03	1.39 (1.03–1.87)	0.04

*Abbreviations:* OR – odds ratio, CI – confidence interval, BMI – body mass index, PE – preeclampsia, FH – family history, MAP - mean arterial blood pressure, HDL – high-density lipoprotein, HbA1c – Hemoglobin A1c, IL-6 – interleukin-6, hs-CRP – high sensitivity C reactive protein, PlGF – inversed placental growth factor.

a Crude p values (logistic regression).

b False discovery rate (FDR) corrected p values are shown for biomarkers (logistic regression).

c Multivariable logistic regression.

d PlGF was inversed, the effect of a lower PlGF (per 1 log_2_ unit) is shown.

**Fig. 2:**
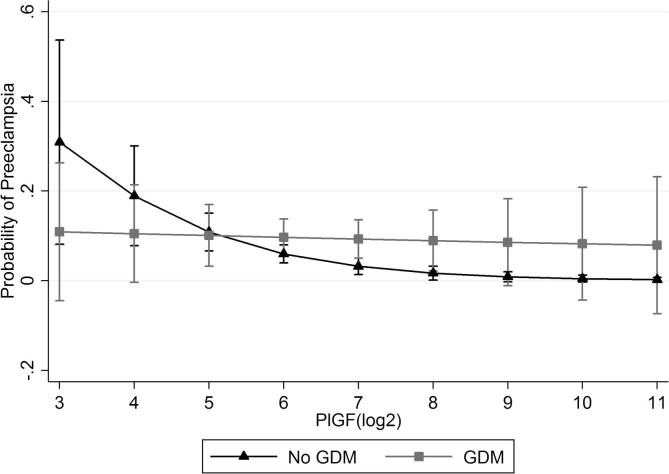
Observed probability of preeclampsia (95% confidence intervals) according to concentration of placental growth factor (PlGF) at 15^+0^–18^+6^ weeks’ gestation in women with obesity with and without gestational diabetes mellitus (GDM).

The prevalence of GDM was 29.6% (n = 241/824). The rate of preeclampsia in women with GDM was 9.5% (n = 23/241) and in women without GDM, 6.2% (n = 36/583). Lower PlGF in the early second trimester increased the odds of preeclampsia only in women without GDM (OR 1.91; 95% CI 1.32–2.78, per log_2_); no association was evident in women with GDM (OR 1.05; 95% CI 0.67–1.63, per log_2_) (Fig. 2). This difference was evident in the interaction test (p = 0.04) (Table 3). Higher IL-6 was associated with preeclampsia in women with GDM (OR 1.85; 95% CI 1.17–2.92, per log_2_) but not in women without GDM (OR 1.25; 95% CI 0.87–1.79, per log_2_), although this was not significant in the interaction test (p = 0.18). Other clinical factors and biomarkers associated with preeclampsia in the full cohort did not differ by GDM status.

**Table 3:** Risk factors for preeclampsia according to GDM status in women with obesity, and interaction test.

	No GDM OR (95% CI) n = 583	GDM OR (95% CI) n = 241	p value a
MAP, per 10 mmHg	2.48 (1.63–3.76)	2.23 (1.30–3.82)	0.76
IL-6, per log_2_ of pg/ml	1.25 (0.87–1.79)	1.85 (1.17–2.92)	0.19
hs-CRP, per log_2_ of mg/L	1.09 (0.81–1.48)	1.21 (0.80–1.83)	0.70
PlGF b, per log_2_ of pg/ml	1.91 (1.32–2.78)	1.05 (0.67–1.63)	0.04

*Abbreviations:* OR – odds ratio, CI – confidence interval, MAP – mean arterial blood pressure, HDL – high-density lipoprotein, IL-6 – interleukin-6, hs-CRP – high sensitivity C reactive protein, PlGF – inversed placental growth factor.

a Likelihood ratio tests for interaction.

b PlGF was inversed; the effect of a lower PlGF (per 1 log_2_ unit) is shown.

The size of associations (odds ratios) and the confidence intervals were broadly similar in the complete case analysis and in the additional sensitivity analyses of imputed cases (Appendix; Table A4) and stratified analyses (Appendix; Table A5), except for nulliparity, which was independently associated with preeclampsia with the increased sample (sensitivity analysis).

## 4 Discussion

Whilst it is widely accepted that the etiology of preeclampsia is heterogeneous, evidence to suggest that risk factors for preeclampsia may differ according to specific high-risk subgroups is relatively new, and presents opportunities for personalized intervention strategies [8], [17]. To our knowledge, there has been no previous attempt to address risk factors for preeclampsia in women with obesity whose pregnancies are also complicated by GDM.

Although we confirmed preeclampsia risk factors previously reported in a heterogeneous BMI population, notably raised mean arterial blood pressure and a reduced concentration of PlGF in early second trimester, we also report that PlGF, increasingly used in prediction or diagnosis of preeclampsia [18], [19], was not associated with preeclampsia in women with obesity who developed GDM. There was, however, a trend for an association between the inflammatory mediator (IL-6) and preeclampsia in women who developed GDM. These data support the hypothesis that different mechanisms may lead to preeclampsia in subgroups of women, and that in obese women who develop both preeclampsia and GDM, PlGF as a predictive/diagnostic test for preeclampsia may have limited use.

Whilst numerous epidemiological studies have demonstrated that obesity increases the risk of preeclampsia, the mechanisms have yet to be fully elucidated [20], [21]. Obesity is characterized by insulin resistance and an exaggerated state of inflammation and oxidative stress which have been implicated in pathways leading to preeclampsia [6], [22]. However, in a recent study of 834 women with obesity (BMI ≥ 30 kg/m^2^) and 3106 lean nulliparous women (BMI 20–25 kg/m^2^) from the international Screening for Pregnancy Endpoints study (SCOPE) and in whom 53 relevant biomarkers were measured, we found no evidence of an association between markers of insulin resistance or inflammatory mediators measured at 14^+0^–16^+0^ weeks’ gestation with later preeclampsia in either lean women or women with obesity [8]. Universal screening for GDM was not performed in SCOPE, which limited the assessment of an interaction of GDM in the association of risk factors at baseline and subsequent development of preeclampsia. In contrast, an OGTT was undertaken in all women included in the present study, enabling examination of interactions between GDM and risk factors for preeclampsia.

The association of low PlGF with preeclampsia, which in the women in this study occurred predominantly at term, confirms our previous observations in women with obesity from the SCOPE cohort [23], but is developed further here by the observation that it is restricted to women with obesity without GDM. Low values of PlGF, or the sFlt-1:PlGF ratio, are increasingly used in the prediction, diagnosis and ‘rule-out’ of preeclampsia [18], [24], [25], [26]. If our observations were to be replicated later in pregnancy, pre-eclampsia diagnostic or rule-out tests using PlGF would require subgroup assessment.

Raised IL-6 has been associated previously with later development of GDM and has been described in preeclampsia at the time of disease in unselected populations [9], [27], [28]. The observed trend between IL-6 (but not hs-CRP) and preeclampsia restricted to women with obesity and GDM warrants further study in larger cohorts [29].

Early second trimester mean arterial blood pressure was strongly associated with preeclampsia in this multi-ethnic heterogeneous population (Appendix; Table A2), confirming many previous reports recognizing that raised blood pressure in early pregnancy is a risk factor for preeclampsia in unselected populations [25], [26].

The strengths of our study include the prospective detailed data collection ensuring almost complete ascertainment of preeclampsia and GDM and the wide range of clinical factors, anthropometric measures and biomarkers available. Another strength is the precise definition of GDM using universal screening with a 75 g OGTT in accordance with IADPSG and WHO recommendations. Measurement of sFlt-1 was not performed as the association with preeclampsia is confined to later pregnancy (after 20 weeks) [30], [31]. Limitations include the 47% (824/1554) of participants excluded because of missing data with the potential for selection bias. However, a sensitivity analysis using multiple imputation which included 82% (1276/1554) of participants provided similar results. We did not perform universal assessment of uterine artery Doppler, a recognized risk factor for preeclampsia, but this reflects current clinical practice [25], [26]. Neither did we explore associations between PlGF later in pregnancy in relation to preeclampsia and GDM. The number of women with a diagnosis of preeclampsia may have limited the power to detect some differences of smaller size. Given the small numbers of cases of preeclampsia within each subgroup these findings, whilst novel, require replication in larger studies. We also acknowledge that the absence of a lean group for comparison in this cohort is a limitation (i.e. no-obesity without GDM, no-obesity with GDM).

## 5 Conclusion

The study of subgroups of women at high risk for preeclampsia has contributed to improved understanding of the heterogeneous etiology of this condition. Our results suggest that the relationship between PlGF and preeclampsia differs in women with obesity according to GDM status, which may indicate different pathways to disease. Studies seeking to translate the use of PlGF as predictive test for preeclampsia into clinical practice should consider sub-group analysis according to obesity status.
