# Supplementary material for: Gestational diabetes modifies the association between PlGF in early pregnancy and preeclampsia in women with obesity
Source: Pregnancy Hypertens. 2018 Jul;13:267–72. doi: 10.1016/j.preghy.2018.07.003 (PMC6130745; doi:10.1016/j.preghy.2018.07.003)
Supplement: Supplementary data 2 [file mmc2.docx]

**Appendix**

Table A1. Analytical methodologies for the biomarkers measured.

| **Biomarker** | **Sample** | **Method** | **Platform** |
| --- | --- | --- | --- |
| Triglycerides | Plasma | Enzymatic, colorimetric | Roche, Cobas c311 |
| HDL cholesterol | Plasma | Homogenous Enzymatic, colorimetric | Roche, Cobas c311 |
| HbA1c | Whole blood | Turbidimetric inhibition immunoassay | Roche, Cobas c311 |
| Adiponectin | Plasma | Enzyme-linked immunosorbent assay | R and D Systems |
| Interleukin-6 | Plasma | Enzyme-linked immunosorbent assay | R and D Systems |
| C-reactive protein | Plasma | Particle enhanced immunoturbidimetric | Roche, Cobas c311 |
| PlGF | Plasma | Fluorescence Immunoassay | Alere, Triage Meter Pro |

Abbreviations: HbA1c - haemoglobin A1c, HDL - high-density lipoprotein, PlGF –placental growth factor.

Table A2. Socio-demographic characteristics of the complete case study population and UPBEAT participants excluded from this analysis.

| **Variable** | **Study participants**  **n= 824**  **Mean (SD) or n (%)** | **Women excluded**  **N=730**  **Mean (SD) or n (%)** | **p value** |
| --- | --- | --- | --- |
| Age | 30.6 (5.4) | 30.3 (5.7) | 0.35 |
| Body Mass Index ^a^ | 35.2 (32.8- 38.5) | 35.1 (32.8- 38.6) | 0.77 |
| Nulliparity | 383 (46.5) | 291 (39.9) | 0.009 |
| Full time education, ≥12 years | 739 (89.7) | 637 (87.3) | 0.13 |
| Maternal ethnicity |  |  |  |
| *Asian* | 54 (6.6) | 41 (5.6) |  |
| *Black* | 154 (18.7) | 247 (33.8) | <0.001 |
| *Other* | 45 (5.5) | 40 (5.5) |  |
| *White* | 571 (69.3) | 402 (55.1) |  |
| Smoking at 15^+0^-18^+6^ weeks' gestation | 55 (6.7) | 53 (7.3) | 0.65 |
| ^a^ Results presented as median (IQR) | | | |

Table A3. Descriptive statistics for clinical risk factors and biomarkers at 15^+0^-18^+6^ weeks according to preeclampsia status in women with obesity.

|  | **No preeclampsia**  **Mean (SD) or n (%)**  **n=765** | **Preeclampsia**  **Mean (SD) or n (%)**  **n=59** |
| --- | --- | --- |
| ***Clinical risk factors*** | |  |
| Age, years | 30.7 (5.4) | 30.0 (4.9) |
| Nulliparous | 348 (45.5) | 35 (59.3) |
| Sum of skinfolds, mm | 121 (26) | 130 (28) |
| Waist, cm | 107.5 (10.2) | 111.2 (12.7) |
| Previous preeclampsia | 27 (3.5) | 5 (8.5) |
| Family history of hypertension | 328 (42.9) | 31 (52.5) |
| MAP, mmHg | 87.7 (7.9) | 93.7 (8.5) |
| ***Biomarkers*** |  |  |
| HDL, per log_2_ of mmol/l | 0.60 (0.47) | 0.46 (0.33) |
| Triglycerides, per log_2_ of mmol/l | 0.74 (0.46) | 0.87 (0.46) |
| HbA1c, mmol | 29.37 (3.76) | 30.43 (4.31) |
| Adiponectin, per log_2_ of ug/ml | 3.35 (0.92) | 3.18 (0.81) |
| IL-6, per log_2_ of pg/ml | 1.57 (0.88) | 1.89 (1.05) |
| hs-CRP, per log_2_ of mg/L | 2.68 (1.14) | 2.85 (1.04) |
| PlGF ^a^, per log_2_ of pg/ml | 6.29 (0.94) | 5.95 (1.02) |

Abbreviations: MAP - mean arterial blood pressure, HDL - high-density lipoprotein, HbA1c - haemoglobin A1c, IL-6 - interleukin-6, hs-CRP - high sensitivity C reactive protein, PlGF - placental growth factor.

^a^ PlGF was inversed; the effect of a lower PlGF (per 1 log_2_ unit) is shown.

Table A4. Sensitivity analysis using multiple imputation (n=1,276): Clinical risk factors and biomarkers at 15^+0^-18^+6^ weeks’ gestation associated with preeclampsia in women with obesity.

|  | **Univariate Analysis**  **OR (95% CI)** | **p value** ^a^ | **Multivariate Analysis**  **OR (95% CI)** | **p value** |
| --- | --- | --- | --- | --- |
| Age, years | 0.98 (0.94 to 1.02) | 0.34 |  |  |
| Body mass index | 1.06 (1.02 to 1.10) | 0.006 | 1.02 (0.95 to 1.09) | 0.64 |
| Nulliparous | 1.88 (1.16 to 3.03) | 0.01 | 1.67 (1.02 to 2.74) | 0.04 |
| Sum of skinfolds, mm | 1.01 (1.01 to 1.02) | 0.02 | 1.01 (1.00 to 1.01) | 0.43 |
| Waist, cm | 1.03 (1.01 to 1.05) | 0.009 | 1.01 (0.98 to 1.04) | 0.69 |
| Previous PE | 3.64 (1.71 to 7.79) | 0.001 |  |  |
| FH of hypertension | 1.53 (0.95 to 2.45) | 0.08 |  |  |
| MAP, per 10 mmHg | 2.46 (1.85 to 3.27) | <0.001 | 2.25 (1.67 to 3.04) | <0.001 |
| HDL, per log_2_ of mmol/l | 0.49 (0.27 to 0.87) | 0.01 |  |  |
| Triglycerides, per log_2_ of mmol/l | 2.09 (1.24 to 3.53) | 0.006 |  |  |
| HbA1c, mmol | 1.08 (1.01 to 1.15) | 0.03 |  |  |
| Adiponectin, per log_2_ of ug/ml | 0.81 (0.62 to 1.05) | 0.11 |  |  |
| IL-6, per log_2_ of pg/ml | 1.46 (1.12 to 1.89) | 0.005 |  |  |
| hs-CRP, per log_2_ of mg/L | 1.17 (0.92 to 1.48) | 0.20 |  |  |
| PlGF ^b^, per log_2_ of pg/ml | 1.41 (1.08 to 1.84) | 0.01 | 1.33 (1.01 to 1.76) | 0.05 |

Abbreviations: OR – odds ratio, CI – confidence interval, PE – preeclampsia, FH – family history, MAP - mean arterial blood pressure, HDL - high-density lipoprotein, HbA1c - Haemoglobin A1c, IL-6 - interleukin-6, hs-CRP - high sensitivity C reactive protein, PlGF - inversed placental growth factor.

^a^ Crude results presented, correction for False Discovery Rate was not performed; ^b^ PlGF was inversed, the effect of a lower PlGF (per 1 log_2_ unit) is shown.

Table A5. Sensitivity analysis using multiple imputation (n=1,276): Risk factors for preeclampsia according to GDM status in women with obesity.

|  | **No GDM**  **OR (95% CI)**  **n=947** | **GDM**  **OR (95% CI)**  **n=329** |
| --- | --- | --- |
| MAP, per 10 mmHg | 2.52 (1.75 to 3.63) | 2.15 (1.33 to 3.48) |
| IL-6, per log_2_ of pg/ml | 1.29 (0.93 to 1.80) | 1.80 (1.16 to 2.78) |
| hs-CRP, per log_2_ of mg/L | 1.11 (0.82 to 1.49) | 1.23 (0.84 to 1.80) |
| PlGF ^a^, per log_2_ of pg/ml | 1.72 (1.22 to 2.43) | 1.12 (0.73 to 1.70) |

Abbreviations: OR – odds ratio, CI – confidence interval, MAP - mean arterial blood pressure, HDL - high-density lipoprotein, IL-6 - interleukin-6, hs-CRP - high sensitivity C reactive protein, PlGF – inversed placental growth factor.

^a^ PlGF was inversed; the effect of a lower PlGF (per 1 log_2_ unit) is shown.
